# Perceived Appropriateness of Assessing for Health-related Socioeconomic Risks Among Adult Patients with Cancer

**DOI:** 10.1158/2767-9764.CRC-22-0283

**Published:** 2023-04-03

**Authors:** Milkie Vu, Kelly Boyd, Emilia H. De Marchis, Bridgette G. Garnache, Laura M. Gottlieb, Cary P. Gross, Nita K. Lee, Stacy Tessler Lindau, Sophia Mun, Victoria A. Winslow, Jennifer A. Makelarski

**Affiliations:** 1 Department of Preventive Medicine, Feinberg School of Medicine, Northwestern University, Chicago, Illinois.; 2 Department of Obstetrics and Gynecology, The University of Chicago Medicine, Chicago, Illinois.; 3 Department of Behavioral, Social, and Health Education Sciences, Rollins School of Public Health, Emory University, Atlanta, Georgia.; 4 Department of Family and Community Medicine, University of California, San Francisco, San Francisco, California.; 5 Cancer Outcomes, Public Policy, and Effectiveness Research (COPPER) Center, Yale School of Medicine, Yale University, New Haven, Connecticut.; 6 National Clinician Scholars Program, Yale University, New Haven, Connecticut.; 7 Department of Medicine-Geriatrics, The University of Chicago Medicine, Chicago, Illinois.; 8 Comprehensive Cancer Center, The University of Chicago Medicine, Chicago, Illinois.; 9 Kaiser Permanente Washington Health Research Institute, Seattle, Washington.; 10 College of Education and Health Services, Benedictine University, Lisle, Illinois.

## Abstract

Cancer treatment can trigger or exacerbate health-related socioeconomic risks (HRSR; food/housing insecurity, transportation/utilities difficulties, and interpersonal violence). The American Cancer Society and National Cancer Institute recommend HRSR screening and referral, but little research has examined the perceptions of patients with cancer on the appropriateness of HRSR screening in healthcare settings. We examined whether HRSR status, desire for assistance with HRSRs, and sociodemographic and health care–related factors were associated with perceived appropriateness of HRSR screening in health care settings and comfort with HRSR documentation in electronic health records (EHR). A convenience sample of adult patients with cancer at two outpatient clinics completed self-administered surveys. We used *χ*^2^ and Fisher exact tests to test for significant associations. The sample included 154 patients (72% female, 90% ages 45 years or older). Thirty-six percent reported ≥1 HRSRs and 27% desired assistance with HRSRs. Overall, 80% thought it was appropriate to assess for HRSRs in health care settings. The distributions of HRSR status and sociodemographic characteristics were similar among people who perceived screening to be appropriate and those who did not. Participants who perceived screening as appropriate were three times as likely to report prior experience with HRSR screening (31% vs. 10%, *P* = 0.01). Moreover, 60% felt comfortable having HRSRs documented in the EHR. Comfort with EHR documentation of HRSRs was significantly higher among patients desiring assistance with HRSRs (78%) compared with those who did not (53%, *P* < 0.01). While initiatives for HRSR screening are likely to be seen by patients with cancer as appropriate, concerns may remain over electronic documentation of HRSRs.

**Significance:**

National organizations recommend addressing HRSRs such as food/housing insecurity, transportation/utilities difficulties, and interpersonal violence among patients with cancer. In our study, most patients with cancer perceived screening for HRSRs in clinical settings as appropriate. Meanwhile, concerns may remain over the documentation of HRSRs in EHRs.

## Introduction

The heavy financial burden of cancer treatment and care in the U.S. forces many patients into financial distress (1–6). National data (1998–2014) estimated that 42% of individuals depleted their life assets in the 2 years following a cancer diagnosis (4). A systematic review found that 12% to 62% of cancer survivors reported being in debt because of their treatment (5). High cancer care costs can trigger or exacerbate socioeconomic hardships (e.g., food insecurity, housing difficulties, transportation needs) in patients with cancer. Socioeconomic hardships are associated with medication nonadherence (7–10), poorer survival rates (11), and worse prognosis and quality of life (12). A 2019 report released by the National Academies of Sciences, Engineering, and Medicine (NASEM) suggested taking social risks into account to improve treatment of acute and chronic illnesses (13). Shortly following the NASEM report's publication, the American Cancer Society (ACS) recommended standard assessment of health-related socioeconomic risks (HRSR) for patients with cancer in health care settings and referral to resources that can address HRSRs (14). The National Cancer Institute (NCI) is prioritizing addressing social risks in cancer care and has released several recent funding and workshop announcements supporting this effort (15).

Past studies have demonstrated that many patients with cancer experience food insecurity (7, 8, 10, 16–21), housing insecurity or worry over rent/mortgage costs (6, 16, 21–23), transportation difficulties (8, 19), utilities concerns (6, 22), and intimate partner violence (24, 25). Because of structural racism (i.e., “the structures, policies, practices, and norms resulting in differential access to the goods, services, and opportunities of society by race”; ref. 26) in the United States, the burden of HRSRs among patients with cancer is also disproportionately concentrated among minoritized racial and ethnic groups (23, 27–29).

Screening tools for HRSRs used in past studies vary in length and focus. Although not specific to patients with cancer, the Accountable Health Communities (AHC) Health-Related Social Needs questionnaire (30) is a standardized screening tool that has been widely adopted and is one of the several tools recommended by the ACS (14). The Center for Medicare and Medicaid Innovation (CMMI) AHC demonstration is using the AHC screening tool to test the impact of community resource referrals for identified needs in 29 bridge organizations across the United States. The project had screened more than 480,000 beneficiaries as of December 2020 (31).

Despite HRSR screening recommendations, it is unclear how patients with cancer perceive the appropriateness of HRSR screening as part of routine clinical care. In non–cancer-specific patient populations, while some studies have found high perceived appropriateness of HRSR screening, some have noted several potential concerns (32–35). Some caregivers of pediatric patients voiced fear of being judged by their providers, embarrassment, and privacy concerns (34, 36). Patients’ relationships (i.e., level of trust) with their providers may also influence perceived appropriateness of HRSR screening (32, 34, 36). Experiences of discrimination are associated with lower perceived appropriateness, while previous experience with HRSR screening in health care settings is associated with higher perceived appropriateness (32). Furthermore, patients may have confidentiality concerns if HRSR screening results are documented in electronic health records (EHR; ref. 32).

It is unclear whether these findings apply to patients with cancer. Cancer treatment is intensive—patients spend a considerable amount of time receiving treatment and recovering from treatment side effects (37–40). Consequently, patients may want to focus on clinical management rather than other issues, especially those perceived as unrelated to medical treatment. Examining factors associated with perceived appropriateness of HRSR screening may help identify subgroups of patients who think screening is inappropriate and inform targeted strategies to address screening concerns.

This study describes patterns and prevalence of HRSRs among people with cancer presenting for care at two outpatient cancer centers. We also analyze relationships between HRSR status, sociodemographic characteristics, and health care–related experiences and (i) desire for assistance with HRSRs; (ii) perceived appropriateness of HRSR screening; and (iii) comfort with HRSR documentation in medical records.

## Materials and Methods

### Eligibility Criteria and Research Procedure

Patients with cancer were recruited from general hematology/oncology, obstetrics and gynecology, breast cancer, and primary care clinics at two NCI Comprehensive Cancer Centers: one in Chicago, Illinois and one in New Haven, Connecticut. The Chicago-based cancer center is located on the South Side of Chicago and in an area with 55% of the population living below 200% of the federal poverty level and 75% identifying as African American or Black (41). The New Haven-based cancer center is located in an area with 64% of the population living below 200% of the federal poverty level and 38% identifying as African American or Black (41). At the time of the study, study participants at the Chicago-based cancer center could receive a list of nearby community resources upon survey completion. The academic medical center that houses the Chicago-based cancer center also operated a food pantry that offered nonperishable food to patients and caregivers, with no eligibility criteria required to receive food or limits on how much food someone could take (42). The New Haven-based cancer center did not operate any program that provided patients with referrals to resources to address HRSRs.

Eligible participants were English- or Spanish-speaking patients with cancer who were ≥18 years of age and able to provide verbal informed consent. We asked participants whether they had ever been told by a doctor or health care professional that they had cancer or any malignancy; anyone responding “Yes” was categorized as a patient with cancer. Research assistants approached potential participants in clinic waiting rooms. Those who expressed interest were provided with information about the study and screened for eligibility. All participants provided verbal consent. Each participant self-completed the survey (total of 50 items) on a tablet computer in either English or Spanish. The survey was translated from English to Spanish by a bilingual Spanish and English-speaking study research assistant. Each participant received $5 as compensation upon survey completion. The research was conducted in accordance with recognized ethical guidelines. The Institutional Review Boards (IRB) of the participating institutions approved this study.

### Measures

Supplementary Data S1 include all study questions and coding instructions for variables.

### HRSR-related Questions

The 10-question AHC screening tool (30, 43) was used to identify five HRSRs: housing insecurity, food insecurity, transportation needs, utility needs, and interpersonal safety concerns. The tool draws on previously validated assessments of these HRSR domains (44). CMMI's instructions for categorizing HRSR status were followed (30).

In addition to the AHC tool, we included several items assessing desire for assistance, perceived appropriateness of HRSR screening, and comfort with having HRSR information entered into the EHR. These additional items were drawn from a previous 10-site study by the Social Interventions Research & Evaluation Network (SIREN; refs. 32, 33). Desire for assistance with HRSRs was assessed by asking whether participants wanted to receive assistance with each of the five HRSRs. Participants were also asked whether it was appropriate to ask about HRSRs at that clinic (“Do you think it is appropriate to be asked these questions about your social and economic needs at this clinic?”), and whether they felt comfortable having their HRSR information documented in their EHR (“Would you be comfortable having these kinds of needs included in your health records, also known as your medical record or chart?”; ref. 32).

### Sociodemographic Characteristics

Sociodemographic data, including participants’ age, gender, race, education, and income were collected via self-report in the survey.

### Previous Experience with HRSR Screening or Assistance

Participants were asked whether they had been screened for or received assistance with any of the five HRSRs in the past 12 months in any health care setting, using measures from the SIREN study (32, 33).

### Experience of Discrimination and Trust in Providers

The Discrimination in Medical Settings scale (45) was administered to ascertain whether participants ever experienced any of seven discrimination or prejudice-related scenarios based on their race, ethnicity, or socioeconomic status when receiving healthcare. Participants who answered affirmatively to any of the seven scenarios were coded as having experienced discrimination. Trust in health care providers (32, 46) was also assessed (scale of 0 to 10, with 0 = Not at all and 10 = Completely). Those who reported a score of 10 out of 10 on the scale were coded as having a high level of trust.

### Data Analysis

We used *χ*^2^ and Fisher exact tests to examine the relationships between sociodemographic characteristics, experience with screening or receiving assistance with HRSRs, discrimination in a medical setting, and trust in providers and: (i) HRSRs (reporting ≥1 HRSRs vs. none), (ii) desire assistance (desiring assistance with any HRSRs vs. none), (iii) perceived appropriateness of HRSR screening (very appropriate/somewhat appropriate vs. neither appropriate nor inappropriate/somewhat inappropriate/very inappropriate), and (iv) comfort with EHR documentation of HRSRs (completely comfortable/somewhat comfortable vs. neither comfortable nor uncomfortable/somewhat uncomfortable/completely uncomfortable). We chose these statistical techniques over the alternative of multivariable logistic regressions for two reasons. First, given the suggested number of events per variable that need to be met to reduce biases in estimates from multivariable logistic regressions (47), we could risk overfitting regressions with our data (48). Second, and critically, our analyses sought to understand the characteristics of those who may be hesitant about HRSR screening and EHR documentation, rather than identifying factors that independently predicted these outcomes.

Three participants with missing data for transportation needs were coded as not having transportation needs. A sensitivity analysis was conducted for perceived appropriateness and comfort with EHR documentation, separating those responding “very” from those responding “somewhat.” The statistical significance criterion was set at 0.05 for all tests. Stata 15.1 (StataCorp LLC) was used for all data analyses.

### Data Availability Statement

The data analyzed in this study are not publicly available due to patient privacy requirements. Data are available upon reasonable request from S.T. Lindau (University of Chicago, Chicago, IL) and C.P. Gross (Yale University, New Haven, CT) and with appropriate IRB approval.

### Ethics Approval and Consent to Participate

All participants provided verbal informed consent. The IRBs of the University of Chicago and Yale University approved this study.

## Results

### Sociodemographic and Health Care Characteristics

Of the 154 patient participants in the sample, 72% were female, 90% were 45 years of age or older (Table 1). More than a quarter (29%) were non-Hispanic African American or Black, and 61% were non-Hispanic White. More than half (58%) had less than a college education. A quarter (27%) had an annual household income of ≤$25,000.

**TABLE 1:** Sociodemographic and health care factors in relation to having ≥1 HRSRs among a sample of patients with cancer

	Total (*N* = 154)		No HRSR (*n* = 98)		≥1 HRSRs (*n* = 56)		
	*n*	%	*n*	%	*n*	%	*P*
Age (*n* = 152)							0.19
18–44	15	9.9	9	9.3	6	10.9	
45–64	64	42.1	36	37.1	28	50.9	
65 or older	73	48.0	52	53.6	21	38.2	
Gender (*n* = 152)							0.76
Female	110	72.4	71	73.2	39	70.9	
Male	42	27.6	26	26.8	16	29.1	
Race (*n* = 151)							<0.001
African American or Black	44	29.1	21	21.6	23	42.6	
White	92	60.9	71	73.2	21	38.9	
Other	15	9.9	5	5.2	10	18.5	
Education (*n* = 154)							.06
Less than a college degree	89	57.8	51	52.0	38	67.9	
College degree or more	65	42.2	47	48.0	18	32.1	
Income (*n* = 124)							<0.001
≤$25,000	33	26.6	4	5.4	29	58.0	
>$25,000	91	73.4	70	94.6	21	42.0	
Previous experience with HRSRs screening (*n* = 154)							0.12
No HRSRs screening	113	73.4	76	77.6	37	66.1	
Any HRSRs screening	41	26.6	22	22.4	19	33.9	
Previous experience with HRSRs assistance (*n* = 154)							<0.001
No HRSRs assistance	134	87.0	93	94.9	41	73.2	
Any HRSRs assistance	20	13.0	5	5.1	15	26.8	
Discrimination in medical settings (*n* = 150)							0.09
No discrimination	122	81.3	82	85.4	40	74.1	
Experienced discrimination	28	18.7	14	14.6	14	25.9	
Trust in health care providers (*n* = 150)							0.19
Less than complete trust	52	34.7	30	30.9	22	41.5	
Complete trust	98	65.3	67	69.1	31	58.5	
Desiring assistance with HRSRs (*n* = 153)							<0.001
No	112	73.2	90	92.8	22	39.3	
Yes	41	26.8	7	7.2	34	60.7	
Appropriateness of HRSRs screening (*n* = 152)							0.53
Not appropriate	31	20.4	22	22.4	9	16.7	
Appropriate	121	79.6	76	77.6	45	83.3	
Comfort with EHR documentation (*n* = 151)							0.32
Uncomfortable	60	39.7	41	42.7	19	34.5	
Comfortable	91	60.3	55	57.3	36	65.5	

Most participants indicated that they had not been screened for HRSRs (73%) or received assistance during medical visits for any HRSRs (87%) in the past 12 months. In addition, 81% reported no experience of discrimination in medical settings and 65% indicated a high level of trust (10 out of 10) in health care providers. Supplementary Data S2 provide additional information on participants from each of the two study sites.

### HRSRs

More than a third of participants screened positive for ≥1 HRSRs (36%; Table 1). More participants with ≥1 HRSRs had lower income (58%) compared with those without HRSRs (5%, *P* < 0.001). Similarly, more participants with ≥1 HRSRs (27%) had previous experience with HRSR assistance in a health care setting compared with those without HRSRs (5%, *P* < 0.001). More participants with ≥1 HRSR identified as African American or Black (43%) compared with those without HRSRs (22%, *P* < 0.001; Table 1).

**FIGURE 1:**
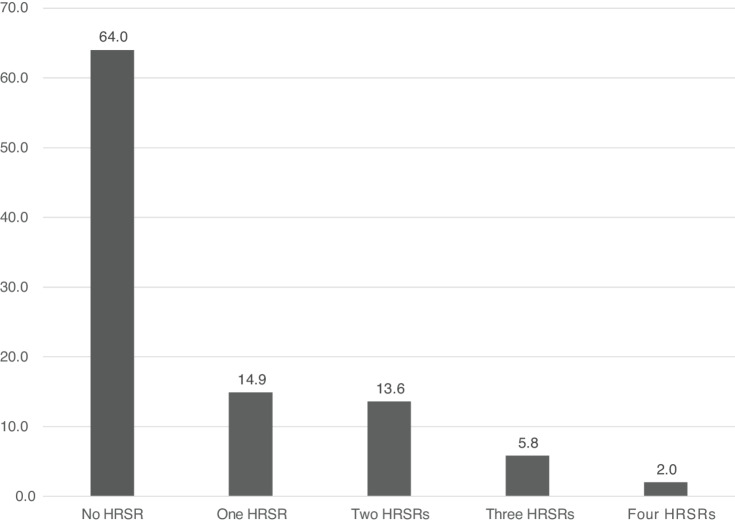
Prevalence of HRSRs among a sample of patients with cancer (*N* = 154).

**FIGURE 2:**
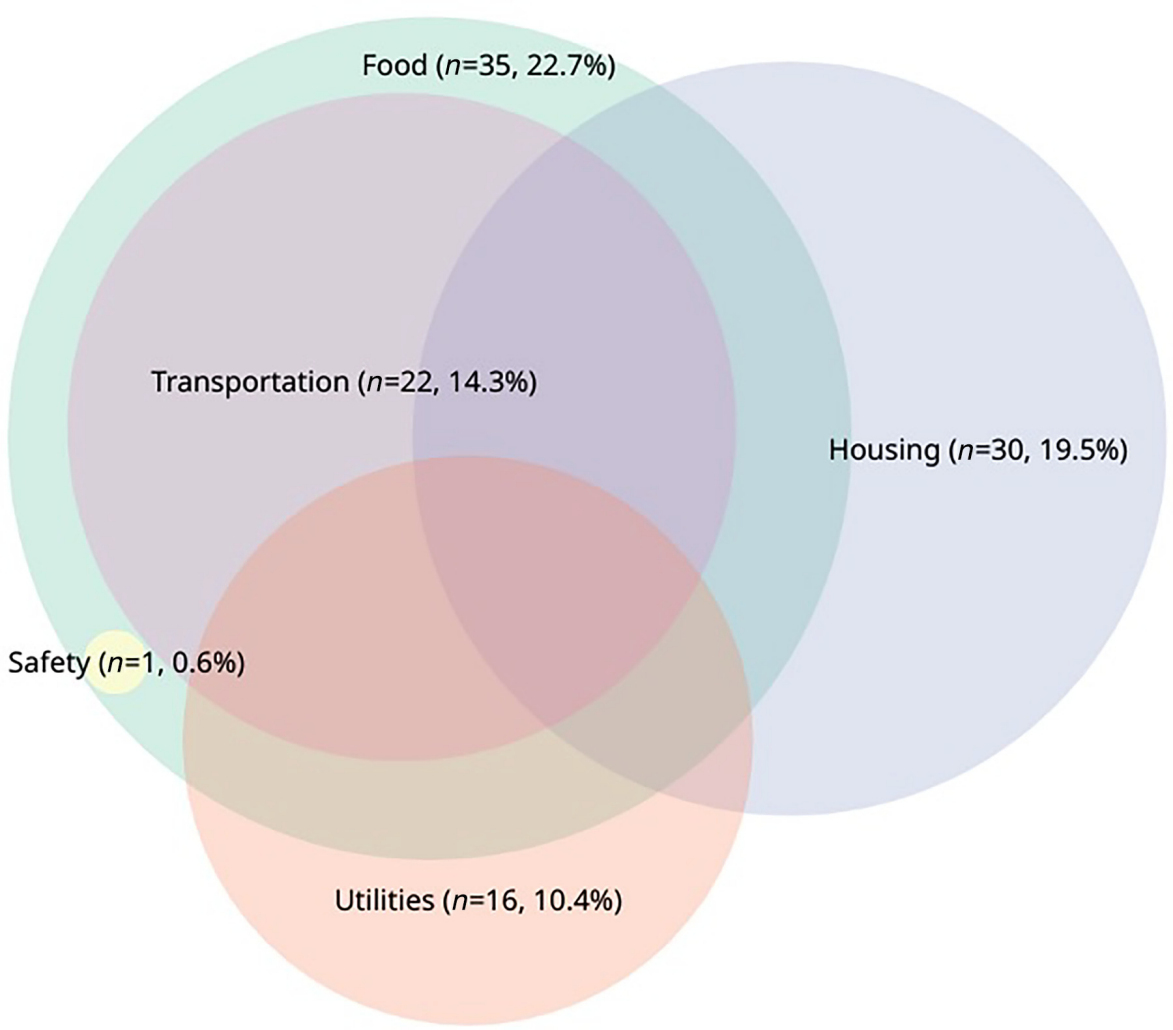
Patterns of overlapping HRSRs among a sample of patients with cancer (*N* = 154). The percentage represents the proportion of the sample with that particular HRSR.

Approximately 21% of participants had ≥2 HRSRs (Fig. 1). Food insecurity was the most prevalent HRSR (23%), followed by housing insecurity (20%), transportation difficulties (14%), utilities concerns (10%), and safety concerns (1%; Fig. 2). All participants who reported transportation difficulties or safety concerns also reported food insecurity (Fig. 2).

### Desire for Assistance with HRSRs

**FIGURE 3:**
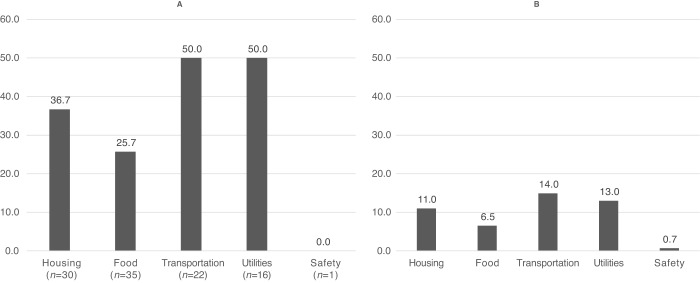
Prevalence of desire for assistance with HRSRs among a sample of patients with cancer. **A**, Among those with the particular HRSR. **B,** Among the entire sample (*N* = 153; one participant had missing data for desire for assistance).

Desire for assistance was several times higher among participants with ≥1 HRSRs (61%) compared with those with none (7%; *P* < 0.001). Half of participants with transportation difficulties (50%) and half with utilities difficulties (50%) desired assistance addressing these HRSRs (Fig. 3). Among the entire sample, 15%, 13%, and 11% desired assistance with transportation difficulties, utilities difficulties, and housing insecurity, respectively (Fig. 3).

Among those with ≥1 HRSRs, a greater proportion of those who desired assistance identified as African American or Black (56% vs. 23%, *P* = 0.03), had lower income (80% vs. 25%, *P* < 0.001), had previous experience with assistance of any of the five HRSRs in a health care setting (41% vs. 5%, *P* < 0.01), and felt comfortable with EHR documentation of HRSRs (79% vs. 43%, *P* < 0.01) than those who did not desire assistance (Table 2). These relationships were similar when the analysis included the entire sample (i.e., both those with and without HRSRs). In addition, in the entire sample, participants who desired assistance had lower educational attainment compared with those not desiring assistance (73% vs. 52%, *P* = 0.02). Desire for assistance did not vary in relation to experience with discrimination or trust in providers.

**TABLE 2:** Sociodemographic and health care factors in relation to desire for assistance with HRSRs among a sample of patients with cancer

	Among those with ≥1 HRSRs							Among the entire sample						
	Total (*N* = 56)		Not desiring assistance (*n* = 22)		Desiring assistance (*n* = 34)			Total (*N* = 153)		Not desiring assistance (*n* = 112)		Desiring assistance (*n* = 41)		
	*N*	%	*n*	%	*n*	%	*P*	*n*	%	*n*	%	*n*	%	*P*
Age							0.39							0.11
18–44	6	10.9	4	18.2	2	6.1		15	9.9	13	11.6	2	5.1	
45–64	28	50.9	10	45.5	18	54.5		64	42.4	42	37.5	22	56.4	
65 or older	21	38.2	8	36.4	13	39.4		72	47.7	57	50.9	15	38.5	
Gender							0.38							0.42
Female	39	70.9	14	63.6	25	75.8		109	72.2	78	70.3	31	77.5	
Male	16	29.1	8	36.4	8	24.2		42	27.8	33	29.7	9	22.5	
Race							0.02							<0.001
African American or Black	23	42.6	5	22.7	18	56.3		44	29.3	22	19.8	22	56.4	
White	21	38.9	10	45.5	11	34.4		91	60.7	77	69.4	14	35.9	
Other	10	18.5	7	31.8	3	9.4		15	10.0	12	10.8	3	7.7	
Education							0.14							0.02
Less than a college degree	38	67.9	12	54.6	26	76.5		88	57.5	58	51.8	30	73.2	
College degree or more	18	32.1	10	45.5	8	23.5		65	42.5	54	48.2	11	26.8	
Income							<0.001							<0.001
≤$25,000	29	58.0	5	25.0	24	80.0		33	26.8	8	9.1	25	71.4	
>$25,000	21	42.0	15	75.0	6	20.0		90	73.2	80	90.9	10	28.6	
Previous experience with HRSRs screening							.56							0.08
No HRSRs screening	37	66.1	16	72.7	21	61.8		113	73.9	87	77.7	26	63.4	
Any HRSRs screening	19	33.9	6	27.3	13	38.2		40	26.1	25	22.3	15	36.6	
Previous experience with HRSRs assistance							<0.01							<0.001
No HRSRs assistance	41	73.2	21	95.5	20	58.8		133	86.9	109	97.3	24	58.5	
Any HRSRs assistance	15	26.8	1	4.5	14	41.2		20	13.1	3	2.7	17	41.5	
Discrimination in medical settings							.53							>0.99
No discrimination	40	74.1	15	68.2	25	78.1		121	81.2	90	81.1	31	81.6	
Experienced discrimination	14	25.9	7	31.8	7	21.9		28	18.8	21	18.9	7	18.4	
Trust in health care providers							.78							0.35
Less than complete trust	22	41.5	9	45.0	13	39.4		52	34.9	36	32.7	16	41.0	
Complete trust	31	58.5	11	55.0	20	60.6		97	65.1	74	67.3	23	59.0	
Appropriateness of HRSRs screening							.27							0.17
Not appropriate	9	16.7	5	25.0	4	11.8		31	20.5	26	23.6	5	12.2	
Appropriate	45	83.3	15	75.0	30	88.2		120	79.5	84	76.4	36	87.8	
Comfort with EHR documentation							<0.01							<0.01
Uncomfortable	19	34.5	12	57.1	7	20.6		60	40.0	51	46.8	9	22.0	
Comfortable	36	65.5	9	42.9	27	79.4		90	60.0	58	53.2	32	78.1	

### Perceived Appropriateness of HRSRs Screening

Overall, 24% and 55% of participants thought it was somewhat or very appropriate to assess for HRSRs at their clinic, respectively (total 80%; Table 3). Distributions of HRSR status, sociodemographic characteristics, experiences of discrimination, and trust in providers were similar among those who perceived screening as appropriate and those who did not. However, prior experience with screening was associated with perceived appropriateness of HRSR screening: among participants who thought screening was somewhat or very appropriate, 31% reported previous HRSR screening experience compared with 10% of participants who did not think screening was appropriate (*P* = 0.01).

**TABLE 3:** Sociodemographic and health care factors in relation to perceived appropriateness of screening and comfort with EHR documentation of HRSRs among a sample of patients with cancer

	Not appropriate (*n* = 31)		Appropriate (*n* = 121)			Uncomfortable (*n* = 60)		Comfortable (*n* = 91)		
	*n*	%	*n*	%	*P*	*n*	%	*n*	%	*P*
Age					.08					0.97
18–44	1	3.2	14	11.8		6	10.2	9	10.0	
45–64	18	58.1	44	37.0		24	40.7	39	43.3	
65 or older	12	38.7	61	51.3		29	49.2	42	46.7	
Gender					.11					0.75
Female	19	61.3	90	75.6		65	72.2	44	74.6	
Male	12	38.7	29	24.4		25	27.8	15	25.4	
Race					.21					0.20
African American or Black	7	22.6	37	31.4		13	21.7	31	35.2	
White	23	74.2	68	57.6		40	66.7	49	55.7	
Other	1	3.2	13	11.0		7	11.7	8	9.1	
Education					.054					0.85
Less than a college degree	13	41.9	74	61.2		34	56.7	53	58.2	
College degree or more	18	58.1	47	38.8		26	43.3	38	41.8	
Income					.12					0.76
≤$25,000	3	13.0	29	29.3		11	25.6	22	28.2	
>$25,000	20	87.0	70	70.7		32	74.4	56	71.8	
Previous experience with HRSRs screening					.01					0.39
No HRSRs screening	28	90.3	83	68.6		46	76.7	64	70.3	
Any HRSRs screening	3	9.7	38	31.4		14	23.3	27	29.7	
Previous experience with HRSRs assistance					.37					0.08
No HRSRs assistance	29	93.5	103	85.1		56	93.3	75	82.4	
Any HRSRs assistance	2	6.5	18	14.9		4	6.7	16	17.6	
Discrimination in medical settings					1.00					>0.99
No discrimination	25	83.3	97	82.2		47	82.5	75	83.3	
Experienced discrimination	5	16.7	21	17.8		10	17.5	15	16.7	
Trust in healthcare providers					.31					0.10
Less than complete trust	13	41.9	38	32.2		25	42.4	26	29.2	
Complete trust	18	58.1	80	67.8		34	57.6	63	70.8	
HRSRs status					.53					0.32
No HRSR	22	71.0	76	62.8		41	68.3	55	60.4	
≥1 HRSRs	9	29.0	45	37.2		19	31.7	36	39.6	
Desiring assistance with HRSRs					.17					<0.01
No	26	83.9	84	70.0		51	85.0	58	64.4	
Yes	5	16.1	36	30.0		9	15.0	32	35.6	

Results from a sensitivity analysis, which separated participants who responded that screening was “very appropriate” from those who responded that it was “somewhat appropriate,” were similar for all comparisons; among those who perceived screening as appropriateness, a higher proportion were of ages 18–44 years compared with those who did not (Supplementary Data S3).

### Comfort with EHR Documentation of HRSRs

Overall, 17% and 43% of participants felt very or somewhat comfortable with having their HRSR information documented in their EHR, respectively (total 60%; Table 3). HRSR status, sociodemographic characteristics, experience of discrimination, and trust in providers were similar among those who felt comfortable with EHR documentation of HRSRs compared with those who did not. However, desire for assistance was associated with comfort with EHR documentation: among participants who reported comfort with EHR documentation, 36% desired assistance with HRSRs compared with only 15% of all participants who were not comfortable with EHR documentation (*P* < 0.01).

In a sensitivity analysis in which those responding “very comfortable” were separated from those responding “somewhat comfortable,” results were similar for all comparisons (Supplementary Data S3).

## Discussion

Among patients with cancer surveyed at two outpatient cancer care sites, 80% reported that it was appropriate to screen for HRSRs and 60% were comfortable with EHR documentation of HRSRs. Perceived appropriateness was higher among patients with previous experience with screening; comfort with EHR documentation was higher among those desiring assistance with HRSRs. We did not identify associations between sociodemographic characteristics, experience of discrimination, or trust in providers and the outcomes of perceived appropriateness and comfort with EHR documentation. Likewise, HRSR status was not associated with either outcome.

The high levels of perceived appropriateness of screening and comfort with EHR documentation are similar to findings reported in the previous 10-site SIREN study in primary care and emergency department patient populations (79% perceived screening as appropriate; 65% felt comfortable with EHR documentation; ref. 32). At the same time, 40% of patients with cancer did not report comfort with EHR documentation of HRSRs. This result suggests that a considerable number may still feel hesitant about electronic documentation of HRSRs, possibly due to concerns about privacy, stigma, or fear that providers may not offer all cancer treatment options based on HRSR status. The positive association between desire for assistance and comfort with EHR documentation could indicate that patients regard EHR documentation as part of a process of getting assistance. Furthermore, our results showed no difference in either perceived appropriateness or comfort with EHR documentation in those with no HRSRs and those with at least one HRSR, which is congruent with results in the previous 10-site study (32).

A higher proportion of those who perceived screening as appropriate had previously been asked about HRSRs at a medical visit (vs. those who did not perceive screening as appropriate). This finding suggests that HRSR screening may feel more appropriate to patients as it becomes more common. We are not aware of prior studies that have examined cancer patient perspectives on clinical implementation of HRSR screening. In general, the interest in adoption of HRSR screening in medical settings in the United States is growing (49), but estimates of adoption vary widely (50). Although hospital executives may have limited understanding of the full range of screening activities, 75% of leaders from 739 hospitals in a 2017 national survey reported that their organization currently screened for at least one of the five HRSRs: food insecurity, housing instability, utilities needs, transportation needs, and interpersonal violence and 24% reported screening for all five (51). Furthermore, as of December 2020, more than 480,000 Medicare & Medicaid beneficiaries in the United States had been screened for HRSRs using the AHC tool (31) in the CMMI AHC demonstration project. A 2017 assessment of 221 NCI Community Oncology Research Program practice groups found that 72% reported screening for financial hardship and 50% had a cancer-specific financial navigator (52). In a 2019 survey of 57 NCI-designated cancer centers, 97% of cancer centers indicated that navigation services were available to help patients apply for financial assistance with transportation, housing, utility bills, and other nonmedical expenses (53).

We found that 39% of patients who screened positive for any HRSR did not indicate a desire for assistance addressing these risks, while 7% of those who did not screen positive for any HRSRs still indicated wanting assistance with addressing HRSRs. This seemingly paradoxical finding has been observed in prior studies (54, 55). It is possible that the AHC tool does not capture all patients with a HRSR, perhaps secondary to poor survey sensitivity (56) or patients’ discomfort reporting HRSRs in health care settings (57). Conversely, the tool may have identified risks that were no longer actively a concern, given that the majority of questions asked about the past 12 months (57).

Given the possibility that screening does not adequately identify all patients with needs, one alternative could be to forgo screening and instead provide HRSR resource information universally to all patients and even caregivers and family members seen in cancer care settings. Or, resource referrals could be targeted to patients who indicate a desire for assistance (rather than on the basis of screening positive for social risks). These approaches could reach patients who are uncomfortable disclosing their HRSR status, those whose risk may be subthreshold (e.g., responses to screening do not trigger a positive result), those who may need these resources in the future, and those who may want the resource information to share with someone else.

Prior studies of the CommunityRx intervention give insight to a universal approach to HRSR intervention. CommunityRx is an automated, EHR-integrated intervention delivered through routine clinical workflows that offers personalized, local community resource information to support self- and social care, caregiving and disease self-management needs (58–60). In a controlled pragmatic trial where CommunityRx was universally offered to all patients, the intervention was found to significantly increase individuals’ self-efficacy or confidence in finding health-promoting community resources (59). Its integration with the EHR was also acceptable to clinicians (58, 61) and about half of patients and clinicians exposed to the community resource information shared it with others (59, 61). While these studies included people with cancer, they were not cancer specific. We acknowledge that even when HRSR screening or resource referral is successfully integrated with clinical care, additional challenges need to be considered. A prior study found that even when technical or logistical barriers were limited, only 7% of patients with social needs ultimately received service to address them (62). In addition, the capacity of local social service systems to respond varies (63). For example, a national study found that while common social risk assessments query housing and transportation difficulties, the capacity of local social service systems to respond to these factors was moderate to low in nearly all U.S. states (63).

Social care studies focused on patients with cancer focus heavily on screening for social risks (i.e., awareness) and connecting patients to social care resources through referral (i.e., assistance; ref. 64). In addition to awareness and assistance, the NASEM's Social and Healthcare Integration Framework (also known as the “5 As Framework”) proposes adjustment, alignment, and advocacy activities to strengthen social and clinical care integration for patients with social risks (13). Adjustment occurs at the patient-clinician level, ensuring that medical recommendations are compatible with patients’ social conditions. Upstream strategies (alignment and advocacy) can occur across multiple sectors. For example, health care systems can collaborate with social services organizations to align health and social care assets and jointly formulate and advance policy to advocate for additional resources and mitigate social risk factors (13). Additional work is needed to inform implementation of these “upstream” strategies to support patients with cancer (64). In addition, there is a need for more systemic solutions to address social risks in the long term.

In the current study, while a higher proportion of patients with cancer who reported HRSRs or a desire for assistance with HRSRs was African American or Black (vs. those who did not report or desire assistance with HRSRs), the distribution of race/ethnicity was similar among those who perceived screening to be appropriate and those who did not. This pattern was similar among those expressing comfort with EHR documentation and those who did not. Incorporating HRSR assessment and assistance in cancer care settings may help to alleviate the disproportionate burden of HRSRs by facilitating receipt of self- and social-care services. Whole person care has been shown to keep patients connected to the health care system and may increase receipt of both cancer treatment (65, 66) and preventive care (67). These mechanisms are important to ongoing efforts to optimize cancer outcomes and reduce racial and ethnic disparities in cancer morbidity and mortality (68).

Study findings should be interpreted in light of several limitations. Although the two sites were selected for the racial/ethnic and socioeconomic diversity of the populations served, the use of convenience sampling can introduce participation bias and limit the generalizability of findings. As our sample is comprised of primarily non-Hispanic White patients and African American or Black patients, results may not be generalizable to patients with cancer from other racial/ethnic groups. The high level of trust in providers among our participants may have limited the generalizability of findings to those with lower trust. This pattern of trust is consistent with a systematic review that found that trust in physicians was strong overall among patients with cancer (69). The small sample size may have resulted in inadequate statistical power to detect some associations. We did not inquire about who would be the most appropriate clinicians to conduct HRSR screening (e.g., physician, nurse, medical assistant, social worker). Other limitations include a lack of data on cancer stage or time since diagnosis, which prevented evaluation of relationships between these factors and outcome variables.

## Conclusions

In this study, a majority of patients with cancer found HRSR screening in healthcare settings appropriate and reported comfort with EHR documentation. Perceived appropriateness and comfort with EHR documentation did not vary based on HRSR status or sociodemographic characteristics. Perceived appropriateness was higher among patients who had previous experiences with HRSR screening in healthcare settings. Comfort with documentation was higher among those who desired assistance with addressing HRSRs. While initiatives for HRSR screening are likely to be seen by patients with cancer as appropriate, concerns may remain over electronic documentation of HRSRs.

## Supplementary Material

Supplementary Data File 1 Survey Questions and Coding Instructions

Supplementary Data File 2 Sociodemographic and healthcare factors in relation to recruitment sites of patients with cancer (N=154)

Supplementary Data File 3 Sociodemographic and healthcare factors in relation to comfort with EHR documentation (N=154)

## References

[1] Carrera PM , Kantarjian HM, Blinder VS. The financial burden and distress of patients with cancer: understanding and stepping-up action on the financial toxicity of cancer treatment. CA Cancer J Clin 2018;68:153–65. 29338071 10.3322/caac.21443 PMC6652174

[2] National Cancer Institute. Financial Toxicity and Cancer Treatment (PDQ®)–Health Professional Version; 2016. Available from: https://www.cancer.gov/about-cancer/managing-care/track-care-costs/financial-toxicity-hp-pdq#_1. 27583328

[3] Zheng Z , Jemal A, Han X, Guy GP Jr, Li C, Davidoff AJ, . Medical financial hardship among cancer survivors in the United States. Cancer 2019;125:1737–47. 30663039 10.1002/cncr.31913

[4] Gilligan AM , Alberts DS, Roe DJ, Skrepnek GH. Death or debt? National estimates of financial toxicity in persons with newly-diagnosed cancer. Am J Med 2018;131:1187–99. 29906429 10.1016/j.amjmed.2018.05.020

[5] Altice CK , Banegas MP, Tucker-Seeley RD, Yabroff KR. Financial hardships experienced by cancer survivors: a systematic review. J Natl Cancer Inst 2017;109:djw205. 27754926 10.1093/jnci/djw205 PMC6075571

[6] Meeker CR , Wong YN, Egleston BL, Hall MJ, Plimack ER, Martin LP, . Distress and financial distress in adults with cancer: an age-based analysis. J Natl Compr Canc Netw 2017;15:1224–33. 28982748 10.6004/jnccn.2017.0161 PMC7569506

[7] Simmons LA , Modesitt SC, Brody AC, Leggin AB. Food insecurity among cancer patients in Kentucky: a pilot study. J Oncol Pract 2006;2:274–9. 20859354 10.1200/jop.2006.2.6.274 PMC2793655

[8] Costas-Muniz R , Leng J, Aragones A, Ramirez J, Roberts N, Mujawar MI, . Association of socioeconomic and practical unmet needs with self-reported nonadherence to cancer treatment appointments in low-income Latino and Black cancer patients. Ethn Health 2016;21:118–28. 25989483 10.1080/13557858.2015.1034658 PMC4653085

[9] Zheng Z , Han X, Guy GP Jr, Davidoff AJ, Li C, Banegas MP, . Do cancer survivors change their prescription drug use for financial reasons? Findings from a nationally representative sample in the United States. Cancer 2017;123:1453–63. 28218801 10.1002/cncr.30560 PMC6080209

[10] McDougall JA , Anderson J, Jaffe SA, Guest DD, Sussman AL, Meisner ALW, . Food insecurity and forgone medical care among cancer survivors. JCO Oncol Pract 2020;16:e922–32. 32384017 10.1200/JOP.19.00736 PMC7489488

[11] Holowatyj AN , Heath EI, Pappas LM, Ruterbusch JJ, Gorski DH, Triest JA, . The epidemiology of cancer among homeless adults in metropolitan detroit. JNCI Cancer Spectr 2019;3:pkz006. 30944890 10.1093/jncics/pkz006 PMC6433093

[12] Hastert TA , McDougall JA, Strayhorn SM, Nair M, Beebe-Dimmer JL, Schwartz AG. Social needs and health-related quality of life among African American cancer survivors: results from the detroit research on cancer survivors study. Cancer 2021;127:467–75. 33225460 10.1002/cncr.33286 PMC7992904

[13] National Academies of Sciences, Engineering, and Medicine. Integrating social care into the delivery of health care: moving upstream to improve the nation's health. Washington, DC: The National Academies Press; 2019. 31940159

[14] Alcaraz KI , Wiedt TL, Daniels EC, Yabroff KR, Guerra CE, Wender RC. Understanding and addressing social determinants to advance cancer health equity in the United States: a blueprint for practice, research, and policy. CA Cancer J Clin 2020;70:31–46. 31661164 10.3322/caac.21586

[15] National Cancer Institute. Addressing social risks in cancer care; 2022. Available from: https://healthcaredelivery.cancer.gov/social-risks/.

[16] Charkhchi P , Dehkordy SF, Carlos RC. Housing and food insecurity, care access, and health status among the chronically ill: an analysis of the behavioral risk factor surveillance system. J Gen Intern Med 2018;33:644–50. 29299816 10.1007/s11606-017-4255-z PMC5910337

[17] Gregory CA , Coleman-Jensen A. Food insecurity, chronic disease, and health among working-age adults, ERR-235, U.S. Department of Agriculture, Economic Research Service; July 2017. Available from: https://www.ers.usda.gov/webdocs/publications/84467/err-235.pdf?v=0.

[18] Gany F , Melnic I, Ramirez J, Wu M, Li Y, Paolantonio L, . Food insecurity among cancer patients enrolled in the supplemental nutrition assistance program (SNAP). Nutr Cancer 2021;73:206–14. 32268803 10.1080/01635581.2020.1743867 PMC8988088

[19] Ayash C , Costas-Muñiz R, Badreddine D, Ramirez J, Gany F. An investigation of unmet socio-economic needs among arab american breast cancer patients compared with other immigrant and migrant patients. J Community Health 2018;43:89–95. 28669006 10.1007/s10900-017-0391-y PMC7010244

[20] Gany F , Lee T, Ramirez J, Massie D, Moran A, Crist M, . Do our patients have enough to eat?: food insecurity among urban low-income cancer patients. J Health Care Poor Underserved 2014;25:1153–68. 25130231 10.1353/hpu.2014.0145 PMC4849892

[21] Zheng Z , Jemal A, Tucker-Seeley R, Banegas MP, Han X, Rai A, . Worry about daily financial needs and food insecurity among cancer survivors in the United States. J Natl Compr Canc Netw 2020;18:315–27. 32135509 10.6004/jnccn.2019.7359

[22] Ko NY , Battaglia TA, Gupta-Lawrence R, Schiller J, Gunn C, Festa K, . Burden of socio-legal concerns among vulnerable patients seeking cancer care services at an urban safety-net hospital: a cross-sectional survey. BMC Health Serv Res 2016;16:196. 27296566 10.1186/s12913-016-1443-1 PMC4906581

[23] Coughlin SS , Datta B. Housing insecurity among cancer survivors: results from the 2017 behavioral risk factor surveillance system survey. J Cancer Policy 2022;31:100320. 35559872 10.1016/j.jcpo.2021.100320

[24] Johnson WA , Pieters HC. Intimate partner violence among women diagnosed with cancer. Cancer Nurs 2016;39:87–96. 25950584 10.1097/NCC.0000000000000265

[25] Coker AL , Follingstad DR, Garcia LS, Bush HM. Intimate partner violence and women's cancer quality of life. Cancer Causes Control 2017;28:23–39. 27943059 10.1007/s10552-016-0833-3 PMC5224925

[26] Jones CP . Confronting institutionalized racism. Phylon 2002;50:7–22.

[27] Fan Q , Keene DE, Banegas MP, Gehlert S, Gottlieb LM, Yabroff KR, . Housing insecurity among patients with cancer. J Natl Cancer Inst 2022;114:1584–92. 36130291 10.1093/jnci/djac136 PMC9949594

[28] American Association for Cancer Research. AACR CANCER DISPARITIES PROGRESS REPORT 2022 Achieving the Bold Vision of Health Equity for Racial and Ethnic Minorities and Other Underserved Populations. Available from: https://cancerprogressreport.aacr.org/wp-content/uploads/sites/2/2022/06/AACR_CDPR_2022.pdf. 10.1158/1055-9965.EPI-20-0269 32938690

[29] Graboyes EM , Chaiyachati KH, Gall JS, Johnson W, Krishnan JA, McManus SS, . Addressing transportation insecurity among patients with cancer. J Natl Cancer Inst 2022;114:1593–600. 36130286 10.1093/jnci/djac134 PMC9745432

[30] The Accountable Health Communities Health-Related Social Needs Screening Tool. Center for Medicare & Medicaid Services. Available from: https://innovation.cms.gov/Files/worksheets/ahcm-screeningtool.pdf.

[31] Brown JA , Berzin O, Clayton M, Cluff L, Derzon J, Evans L, . Accountable health communities (AHC) model evaluation, first evaluation report. RTI International; Center for Medicare & Medicaid Innovation; 2020. p. 1–72. Available from: https://innovation.cms.gov/data-and-reports/2020/ahc-first-eval-rpt.

[32] De Marchis EH , Hessler D, Fichtenberg C, Adler N, Byhoff E, Cohen AJ, . Part I: a quantitative study of social risk screening acceptability in patients and caregivers. Am J Prev Med 2019;57:S25–37. 31753277 10.1016/j.amepre.2019.07.010 PMC7336892

[33] Byhoff E , De Marchis EH, Hessler D, Fichtenberg C, Adler N, Cohen AJ, . Part II: a qualitative study of social risk screening acceptability in patients and caregivers. Am J Prev Med 2019;57:S38–46. 31753278 10.1016/j.amepre.2019.07.016 PMC6876708

[34] Knowles M , Khan S, Palakshappa D, Cahill R, Kruger E, Poserina BG, . Successes, challenges, and considerations for integrating referral into food insecurity screening in pediatric settings. J Health Care Poor Underserved 2018;29:181–91. 29503293 10.1353/hpu.2018.0012

[35] Cullen W , Gulati G, Kelly BD. Mental health in the Covid-19 pandemic. QJM 2020;113:311–2. 32227218 10.1093/qjmed/hcaa110 PMC7184387

[36] Cullen D , Attridge M, Fein JA. Food for thought: a qualitative evaluation of caregiver preferences for food insecurity screening and resource referral. Acad Pediatr 2020;20:1157–62. 32302758 10.1016/j.acap.2020.04.006 PMC9524403

[37] Hall ET , Sridhar D, Singhal S, Fardeen T, Lahijani S, Trivedi R, . Perceptions of time spent pursuing cancer care among patients, caregivers, and oncology professionals. Support Care Cancer 2021;29:2493–500. 32935204 10.1007/s00520-020-05763-9

[38] Yabroff KR , Davis WW, Lamont EB, Fahey A, Topor M, Brown ML, . Patient time costs associated with cancer care. J Natl Cancer Inst 2007;99:14–23. 17202109 10.1093/jnci/djk001

[39] Bange EM , Doucette A, Gabriel PE, Porterfield F, Harrigan JJ, Wang R, . Opportunity costs of receiving palliative chemotherapy for metastatic pancreatic ductal adenocarcinoma. JCO Oncol Pract 2020;16:e678–87. 32130074 10.1200/JOP.19.00328 PMC7427417

[40] Gupta A , Eisenhauer EA, Booth CM. The time toxicity of cancer treatment. J Clin Oncol 2022;40:1611–5. 35235366 10.1200/JCO.21.02810

[41] US Census Bureau. 2019 American Community Survey Data (1-year estimates); 2019. Available from: https://data.census.gov/cedsci/

[42] Frazier CRM , Pinkerton EA, Grana M, Davis M, Asay S, Makelarski JA, . Feed1st, no questions asked: how a hospital-based food pantry program grew its impact during the COVID-19 pandemic. Am J Public Health 2022;112:1394–8. 36007206 10.2105/AJPH.2022.306984 PMC9480483

[43] Billioux A , Verlander K, Anthony S, Alley D. Standardized screening for health-related social needs in clinical settings: the Accountable Health Communities screening tool. Washington, D.C.: National Academy of Medicine; 2017. Available from: https://nam.edu/wp-content/uploads/2017/05/Standardized-Screening-for-Health-Related-Social-Needs-in-Clinical-Settings.pdf.

[44] O'Gurek DT , Henke C. A practical approach to screening for social determinants of health. Fam Pract Manag 2018;25:7–12. 29989777

[45] Peek ME , Nunez-Smith M, Drum M, Lewis TT. Adapting the everyday discrimination scale to medical settings: reliability and validity testing in a sample of African American patients. Ethn Dis 2011;21:502–9. 22428358 PMC3350778

[46] Agency for Healthcare Research and Quality. About the CAHPS® Cultural Competence Item Set; 2012. Available from: https://theinstitute.umaryland.edu/media/ssw/institute/national-center-documents/Consumer-Assessment-for-Healthcare-and-Provider-Systems-CAHPS-Cultural-Competence-Data-Set.pdf

[47] Peduzzi P , Concato J, Kemper E, Holford TR, Feinstein AR. A simulation study of the number of events per variable in logistic regression analysis. J Clin Epidemiol 1996;49:1373–9. 8970487 10.1016/s0895-4356(96)00236-3

[48] Stoltzfus JC . Logistic regression: a brief primer. Acad Emerg Med 2011;18:1099–104. 21996075 10.1111/j.1553-2712.2011.01185.x

[49] Kreuter MW , Thompson T, McQueen A, Garg R. Addressing social needs in health care settings: evidence, challenges, and opportunities for public health. Annu Rev Public Health 2021;42:329–44. 33326298 10.1146/annurev-publhealth-090419-102204 PMC8240195

[50] Cartier Y , Gottlieb L. The prevalence of social care in US health care settings depends on how and whom you ask. BMC Health Serv Res 2020;20:481. 32471466 10.1186/s12913-020-05338-8 PMC7260787

[51] Fraze TK , Brewster AL, Lewis VA, Beidler LB, Murray GF, Colla CH. Prevalence of screening for food insecurity, housing instability, utility needs, transportation needs, and interpersonal violence by US physician practices and hospitals. JAMA Netw Open 2019;2:e1911514. 31532515 10.1001/jamanetworkopen.2019.11514 PMC6752088

[52] McLouth LE , Nightingale CL, Dressler EV, Snavely AC, Hudson MF, Unger JM, . Current practices for screening and addressing financial hardship within the National Cancer Institute's community oncology research program. Cancer Epidemiol Biomarkers Prev 2021;30:669–75. 33355237 10.1158/1055-9965.EPI-20-1157 PMC8026561

[53] de Moor JS , Mollica M, Sampson A, Adjei B, Weaver SJ, Geiger AM, . Delivery of financial navigation services within national cancer institute–designated cancer centers. JNCI Cancer Spectr 2021;5:pkab033. 34222790 10.1093/jncics/pkab033 PMC8242138

[54] Bottino CJ , Rhodes ET, Kreatsoulas C, Cox JE, Fleegler EW. Food Insecurity screening in pediatric primary care: can offering referrals help identify families in need? Acad Pediatr 2017;17:497–503. 28302365 10.1016/j.acap.2016.10.006

[55] De Marchis EH , Hessler D, Fichtenberg C, Fleegler EW, Huebschmann AG, Clark CR, . Assessment of social risk factors and interest in receiving health care-based social assistance among adult patients and adult caregivers of pediatric patients. JAMA Netw Open 2020;3:e2021201. 33064137 10.1001/jamanetworkopen.2020.21201 PMC7568201

[56] Henrikson NB , Blasi PR, Dorsey CN, Mettert KD, Nguyen MB, Walsh-Bailey C, . Psychometric and pragmatic properties of social risk screening tools: a systematic review. Am J Prev Med 2019;57:S13–24. 31753276 10.1016/j.amepre.2019.07.012

[57] Marchis EHD , Alderwick H, Gottlieb LM. Do patients want help addressing social risks? J Am Board Fam Med 2020;33:170–5. 32179597 10.3122/jabfm.2020.02.190309

[58] Lindau ST , Makelarski J, Abramsohn E, Beiser DG, Escamilla V, Jerome J, . CommunityRx: a population health improvement innovation that connects clinics to communities. Health Aff 2016;35:2020–9. 10.1377/hlthaff.2016.0694 PMC5573228 27834242

[59] Lindau ST , Makelarski J, Abramsohn E, Beiser DG, Boyd K, Chou C, . CommunityRx: a real-world controlled clinical trial of a scalable, low-intensity community resource referral intervention. Am J Public Health 2019;109:600–6. 30789775 10.2105/AJPH.2018.304905 PMC6417580

[60] Tung EL , Abramsohn EM, Boyd K, Makelarski JA, Beiser DG, Chou C, . Impact of a low-intensity resource referral intervention on patients’ knowledge, beliefs, and use of community resources: results from the communityrx trial. J Gen Intern Med 2020;35:815–23. 10.1007/s11606-019-05530-5 PMC7080911 31749028

[61] Lindau ST , Makelarski JA, Abramsohn EM, Beiser DG, Boyd K, Huang ES, . Sharing information about health-related resources: observations from a community resource referral intervention trial in a predominantly African American/Black community. J Assoc Inf Sci Technol 2021;73:438–48. 10.1002/asi.24560 PMC12395496 40893821

[62] Wallace AS , Luther BL, Sisler SM, Wong B, Guo JW. Integrating social determinants of health screening and referral during routine emergency department care: evaluation of reach and implementation challenges. Implement Sci Commun 2021;2:114. 34620248 10.1186/s43058-021-00212-y PMC8499465

[63] Kreuter M , Garg R, Thompson T, McQueen A, Javed I, Golla B, . Assessing the capacity of local social services agencies to respond to referrals from health care providers. Health Aff 2020;39:679–88. 10.1377/hlthaff.2019.01256 PMC8336061 32250682

[64] Sanchez JI , Adjei BA, Randhawa G, Medel J, Doose M, Oh A, . National cancer institute–funded social risk research in cancer care delivery: opportunities for future research. J Natl Cancer Inst 2022;114:1628–35. 36073952 10.1093/jnci/djac171 PMC9949593

[65] Gany F , Melnic I, Wu M, Li Y, Finik J, Ramirez J, . Food to overcome outcomes disparities: a randomized controlled trial of food insecurity interventions to improve cancer outcomes. J Clin Oncol 2022;40:3603–12. 35709430 10.1200/JCO.21.02400 PMC9622577

[66] Etminani-Ghasrodashti R , Kan C, Mozaffarian L. Investigating the role of transportation barriers in cancer patients’ decision making regarding the treatment process. Transp Res Rec 2021;2675:175–87.

[67] Yue D , Pourat N, Chen X, Lu C, Zhou W, Daniel M, . Enabling services improve access to care, preventive services, and satisfaction among health center patients. Health Aff 2019;38:1468–74. 10.1377/hlthaff.2018.05228 31479374

[68] Islami F , Guerra CE, Minihan A, Yabroff KR, Fedewa SA, Sloan K, . American Cancer Society's report on the status of cancer disparities in the United States, 2021. CA Cancer J Clin 2022;72:112–43. 34878180 10.3322/caac.21703

[69] Hillen MA , de Haes H, Smets EMA. Cancer patients’ trust in their physician—a review. Psychooncology 2011;20:227–41. 20878840 10.1002/pon.1745

