# Supplementary material for: Perceived Appropriateness of Assessing for Health-related Socioeconomic Risks Among Adult Patients with Cancer
Source: Cancer Res Commun. 2023 Apr 3;3(4):521–31. doi: 10.1158/2767-9764.CRC-22-0283 (PMC10069714; doi:10.1158/2767-9764.CRC-22-0283)
Supplement: Supplementary Data File 1 — Survey Questions and Coding Instructions [file crc-22-0283-s01.docx]

**Supplementary Data File A. Survey Questions and Coding Instructions**

Coding instructions appear in red text.

**Health-Related Social Risks (HRSRs)**

Q1. What is your living situation today?

1. I have a steady place to live
2. I have a place to live today, but I am worried about losing it in the future
3. I do not have a steady place to live (I am temporarily staying with others, in a hotel, in a shelter, living outside on the street, on a beach, in a car, abandoned building, bus or train station, or in a park).

Q2. Think about the place you live. Do you have problems with any of the following? (Check all that apply)

1. Pests such as bugs, ants, or mice
2. Mold
3. Lead paint or pipes
4. Lack of heat
5. Oven or stove not working
6. Smoke detectors missing or not working
7. Water leaks
8. None of the above

Participants were coded as experiencing housing instability if they either

1) chose option b or c for Q1

or

2) chose any option of a through g for Q2

Some people have made the following statements about their food situation. Please answer whether the statements were OFTEN, SOMETIMES, or NEVER true for you and your household in the last 12 months.

Q3. Within the past 12 months, you worried that your food would run out before you got money to buy more.

1. Often true
2. Sometimes true
3. Never true

Q4. Within the past 12 months, the food you bought just didn't last and you didn't have money to get more.

1. Often true
2. Sometimes true
3. Never true

Participants are coded as experiencing food insecurity if they either

1) chose option a or b for Q3

or

2) chose option a or b for Q4

Q5. In the past 12 months, has lack of reliable transportation kept you from medical appointments, meetings, work or from getting things needed for daily living?

1. Yes
2. No

Participants were coded as experiencing transportation difficulties if they chose option b for Q5.

Q6. In the past 12 months has the electric, gas, oil, or water company threatened to shut off services in your home?

1. No
2. Yes
3. Already shut off

Participants were coded as experiencing utilities needs if they chose either option (b) or (c) for Q6.

Because violence and abuse happens to a lot of people and affects their health, we are asking the following questions.

Q7. How often does anyone, including family and friends, physically hurt you?

1. Never
2. Rarely
3. Sometimes
4. Fairly often
5. Frequently

Q8. How often does anyone, including family and friends, insult or talk down to you?

1. Never
2. Rarely
3. Sometimes
4. Fairly often
5. Frequently

Q9. How often does anyone, including family and friends, threaten you with harm?

1. Never
2. Rarely
3. Sometimes
4. Fairly often
5. Frequently

Q10. How often does anyone, including family and friends, scream or curse at you?

1. Never
2. Rarely
3. Sometimes
4. Fairly often
5. Frequently

For Q7 through Q10, each answer option was scored sequentially from 0 to 5 points, where “never” = 0 point and “frequently” = 5 points. Participants were coded as experiencing a risk of violence if the total score for the four questions (Q7 to Q10) was greater than 7.

**Desire for Assistance with HRSRs**

Q11. Would you like to receive assistance with any of the issues below: (check all that apply)

1. Housing
2. Food access
3. Medical or non-medical transportation
4. Electric, gas, oil, or water utility services
5. Your safety, or violence in your household
6. None of these

Participants were coded as desiring help with HRSRs if they chose any option a through e for Q11.

**Perceived Appropriateness of HRSRs Screening**

Q12. Do you think it is appropriate to be asked these questions about your social and economic needs at this clinic?

1. Very appropriate
2. Somewhat appropriate
3. Neither appropriate nor inappropriate
4. Somewhat inappropriate
5. Very inappropriate

Participants were coded as perceiving HRSRs screening as appropriate if they chose option a or b for Q12.

**Comfort with EHR Documentation of HRSRs**

Q13. Would you be comfortable having these kinds of needs included in your health records (also known as your medical record or chart)?

1. Completely comfortable
2. Somewhat comfortable
3. Neither comfortable nor uncomfortable
4. Somewhat uncomfortable
5. Completely uncomfortable

Participants were coded as feeling comfortable with EHR documentation of HRSRs if they chose option a or b for Q13.

**Sociodemographic Characteristics**

Q14. What is your age?

1. 18 to 24
2. 25 to 34
3. 35 to 44
4. 45 to 54
5. 55 to 64
6. 65 to 74
7. 75 or older

Responses for Q14 were grouped into three categories: 18 to 44 (option a through c), 45 to 64 (option d and e), and 65 and above (option f and g).

Q15. What is the highest grade (or year) of school you completed?

1. Elementary School: 1 year
2. Elementary School: 2 years
3. Elementary School: 3 years
4. Elementary School: 4 years
5. Elementary School: 5 years
6. Elementary School: 6 years
7. Elementary School: 7 years
8. Elementary School: 8 years
9. High School: 9 years
10. High School: 10 years
11. High School: 11 years
12. High School: 12 years
13. College: 13 years
14. College: 14 years
15. College: 15 years
16. College: 16 years
17. Graduate School: 17 years
18. Graduate School: 18 years
19. Graduate School: 19 years
20. Graduate School: 20+ years

Responses were grouped into two categories: Less than a college degree (option a through o) and and College degree or more (option p through t).

Q16. How do you describe your gender?

1. Male
2. Female
3. Trans male/Trans man
4. Trans female/Trans woman
5. Gender-queer/Gender non-conforming
6. Different identity
7. Prefer not to answer

For Q16, no participant chose option c, d, e, f, or g. Responses were grouped into two categories for Q16: Male (option a) or Female (option b).

Q17. What is your race? (Mark all that apply)

1. White
2. Black or African American
3. American Indian or Alaska Native
4. Asian Indian
5. Chinese
6. Filipino
7. Japanese
8. Korean
9. Vietnamese
10. Native Hawaiian
11. Guamanian or Chamorro
12. Samoan
13. Other Pacific Islander
14. Other Asian
15. Some other race

Responses for Q17 were grouped into three categories: White (if participant only chose option a), Black or African-American (if participant only chose option b), or Other (if participant only chose any option of d through o or if they chose more than one option)

Q18. Which of the following categories best describes your total combined household income for the past 12 months?

1. 0 - $5,000
2. $5,001 - $10,000
3. $10,001 - $15,000
4. $15,001 - $20,000
5. $20,001 - $25,000
6. $25,001 - $30,000
7. $30,001 - $35,000
8. $35,001 - $40,000
9. $40,001 - $50,000
10. $50,001 - $75,000
11. $75,001 - $100,000
12. $100,001 - $150,000
13. $150,000+
14. Don't know
15. Would rather not say

Responses for Q18 were grouped into two categories: ≤ $25,000 (option a through e) and >$25,000 (option f through m).

**Other Healthcare-Related Factors**

When getting health care, have you ever had any of the following things happen to you because of your race, ethnicity, or socioeconomic status?

Q19. Felt like a doctor or nurse was not listening to what you were saying?

1. Yes
2. No

Q20. Treated you with less respect than other people?

1. Yes
2. No

Q21. Received poorer services than other people?

1. Yes
2. No

Q22. Treated with less courtesy than other people?

1. Yes
2. No

Q23. Had a doctor or nurse act as if he or she was better than you?

1. Yes
2. No

Q24. Had a doctor or nurse act as if he or she thinks you were not smart?

1. Yes
2. No

Q25. Had a doctor or nurse act as if he or she was afraid of you?

1. Yes
2. No

Participants were coded as having experienced discrimination in medical settings if they chose option a for any questions of Q19 through Q25.

Q26. How much do you trust your health care provider(s) at this clinic?

|  | Not at all | Completely |
| --- | --- | --- |

|  |  | 0 | 1 | 2 | 3 | 4 | 5 | 6 | 7 | 8 | 9 | 10 |
| --- | --- | --- | --- | --- | --- | --- | --- | --- | --- | --- | --- | --- |

| 1 () | 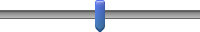 |
| --- | --- |

Participants were coded as having “Less than complete trust” if they chose any option of 0 through 9 and as having “Complete trust” if they chose option 10.

Q27. In the last 12 months, not including today, have you been asked about any of the following in any health care setting: (check all that apply)

1. Yes, Housing
2. Yes, Food access
3. Yes, Medical or non-medical transportation
4. Yes, Electric, gas, oil, or water utility services
5. Yes, Your safety, or violence in your household
6. No, None of these

Participants were coded as having been previously screened for HRSRs in healthcare settings if they chose any of the options of a through e for Q27.

Q28. In the last 12 months, not including today, have you received assistance from anyone in any health care setting related to: (check all that apply)

1. Housing
2. Food access
3. Medical or non-medical transportation
4. Electric, gas, oil, or water utility services
5. Your safety, or violence in your household
6. None of these

Participants were coded as having previously received assistance for HRSRs in healthcare settings if they chose any of the options of a through e for Q28.
