# Supplementary material for: Perceived Appropriateness of Assessing for Health-related Socioeconomic Risks Among Adult Patients with Cancer
Source: Cancer Res Commun. 2023 Apr 3;3(4):521–31. doi: 10.1158/2767-9764.CRC-22-0283 (PMC10069714; doi:10.1158/2767-9764.CRC-22-0283)
Supplement: Supplementary Data File 2 — Sociodemographic and healthcare factors in relation to recruitment sites of patients with cancer (N=154) [file crc-22-0283-s02.docx]

| **Supplementary Data File B. Sociodemographic and healthcare factors in relation to recruitment sites of patients with cancer (N=154)** | | | | | | | |
| --- | --- | --- | --- | --- | --- | --- | --- |
|  | Total  (N=154) | | Chicago site  (n=109) | | New Haven site  (n=45) | | p-value |
|  | n | % | n | % | N | % |  |
| Age (n=152) |  |  |  |  |  |  | .97 |
| 18-44 | 15 | 9.9 | 11 | 10.2 | 4 | 9.1 |  |
| 45-64 | 64 | 42.1 | 46 | 42.6 | 18 | 40.9 |  |
| 65 or older | 73 | 48.0 | 51 | 47.2 | 22 | 50.0 |  |
| Gender (n=152) |  |  |  |  |  |  | .01 |
| Female | 110 | 72.4 | 71 | 66.4 | 39 | 86.7 |  |
| Male | 42 | 27.6 | 36 | 33.6 | 6 | 13.3 |  |
| Race (n=151) |  |  |  |  |  |  | .10 |
| African American or Black | 44 | 29.1 | 36 | 34.0 | 8 | 17.8 |  |
| White | 92 | 60.9 | 59 | 55.7 | 33 | 73.3 |  |
| Other | 15 | 9.9 | 11 | 10.4 | 4 | 8.9 |  |
| Education (n=154) |  |  |  |  |  |  | .47 |
| Less than a college degree | 89 | 57.8 | 65 | 59.6 | 24 | 53.3 |  |
| College degree or more | 65 | 42.2 | 44 | 40.4 | 21 | 46.7 |  |
| Income (n=124) |  |  |  |  |  |  | .35 |
| ≤ $25,000 | 33 | 26.6 | 27 | 29.0 | 6 | 19.4 |  |
| >$25,000 | 91 | 73.4 | 66 | 71.0 | 25 | 80.7 |  |
| Previous experience with HRSR screening (n=154) |  |  |  |  |  |  | .55 |
| No HRSRs screening | 113 | 73.4 | 78 | 71.6 | 35 | 77.8 |  |
| Any HRSRs screening | 41 | 26.6 | 31 | 28.4 | 10 | 22.2 |  |
| Previous experience with HRSR assistance (n=154) |  |  |  |  |  |  | .43 |
| No HRSRs assistance | 134 | 87.0 | 93 | 85.3 | 41 | 91.1 |  |
| Any HRSRs assistance | 20 | 13.0 | 16 | 14.7 | 4 | 8.9 |  |
| Discrimination in medical settings (n=150) |  |  |  |  |  |  | .07 |
| No discrimination | 122 | 81.3 | 81 | 77.1 | 41 | 91.1 |  |
| Experienced discrimination | 28 | 18.7 | 24 | 22.9 | 4 | 8.9 |  |
| Trust in healthcare providers (n=150) |  |  |  |  |  |  | .02 |
| Less than complete trust | 52 | 34.7 | 43 | 40.6 | 9 | 20.5 |  |
| Complete trust | 98 | 65.3 | 63 | 59.4 | 35 | 79.6 |  |
| HRSRs status |  |  |  |  |  |  | .048 |
| No HRSR | 98 | 63.6 | 64 | 58.7 | 34 | 75.6 |  |
| ≥1 HRSRs | 56 | 36.4 | 45 | 41.3 | 11 | 24.4 |  |
| Desiring assistance with HRSRs (n=153) |  |  |  |  |  |  | .55 |
| No | 112 | 73.2 | 77 | 71.3 | 35 | 77.8 |  |
| Yes | 41 | 26.8 | 31 | 28.7 | 10 | 22.2 |  |
| Appropriateness of HRSRs screening (n=152) |  |  |  |  |  |  | .51 |
| Not appropriate | 31 | 20.4 | 24 | 22.2 | 7 | 15.9 |  |
| Appropriate | 121 | 79.6 | 84 | 77.8 | 37 | 84.1 |  |
| Comfort with EHR documentation (n=151) |  |  |  |  |  |  | .85 |
| Uncomfortable | 60 | 39.7 | 42 | 39.3 | 18 | 40.9 |  |
| Comfortable | 91 | 60.3 | 65 | 60.8 | 26 | 59.1 |  |
