# Supplementary material for: Perceived Appropriateness of Assessing for Health-related Socioeconomic Risks Among Adult Patients with Cancer
Source: Cancer Res Commun. 2023 Apr 3;3(4):521–31. doi: 10.1158/2767-9764.CRC-22-0283 (PMC10069714; doi:10.1158/2767-9764.CRC-22-0283)
Supplement: Supplementary Data File 3 — Sociodemographic and healthcare factors in relation to comfort with EHR documentation (N=154) [file crc-22-0283-s03.docx]

| **Supplementary Data File C. Sociodemographic and healthcare factors in relation to comfort with EHR documentation (N=154)** | | | | | | | | | |
| --- | --- | --- | --- | --- | --- | --- | --- | --- | --- |
|  | Total  (N=154) | | Not comfortable  (n=60) | | Somewhat comfortable  (n=26) | | Very comfortable  (n=65) | | p-value |
|  | n | % | n | % | N | % | N | % |  |
| Age (n=152) |  |  |  |  |  |  |  |  | .50 |
| 18-44 | 15 | 10.1 | 6 | 10.2 | 5 | 19.2 | 4 | 6.3 |  |
| 45-64 | 63 | 42.3 | 24 | 40.7 | 10 | 38.5 | 29 | 45.3 |  |
| 65 or older | 71 | 47.7 | 29 | 49.2 | 11 | 42.3 | 31 | 48.4 |  |
| Gender (n=152) |  |  |  |  |  |  |  |  | .09 |
| Female | 109 | 73.2 | 44 | 74.6 | 14 | 56.0 | 51 | 78.5 |  |
| Male | 40 | 26.9 | 15 | 25.4 | 11 | 44.0 | 14 | 21.5 |  |
| Race (n=151) |  |  |  |  |  |  |  |  | .20 |
| African American or Black | 44 | 29.7 | 13 | 21.7 | 8 | 34.8 | 23 | 35.4 |  |
| White | 89 | 60.1 | 40 | 66.7 | 11 | 47.8 | 38 | 58.5 |  |
| Other | 15 | 10.1 | 7 | 11.7 | 4 | 17.4 | 4 | 6.2 |  |
| Education (n=154) |  |  |  |  |  |  |  |  | .85 |
| Less than a college degree | 87 | 57.6 | 34 | 56.7 | 14 | 53.9 | 39 | 60.0 |  |
| College degree or more | 64 | 42.4 | 26 | 43.3 | 12 | 46.2 | 26 | 40.0 |  |
| Income (n=124) |  |  |  |  |  |  |  |  | .48 |
| ≤ $25,000 | 33 | 27.3 | 11 | 25.6 | 9 | 37.5 | 13 | 24.1 |  |
| >$25,000 | 88 | 72.7 | 32 | 74.4 | 15 | 62.5 | 41 | 75.9 |  |
| Previous experience with HRSR screening (n=154) |  |  |  |  |  |  |  |  | .71 |
| No HRSRs screening | 110 | 72.9 | 46 | 76.7 | 18 | 69.2 | 46 | 70.8 |  |
| Any HRSRs screening | 41 | 27.2 | 14 | 23.3 | 8 | 30.8 | 19 | 29.2 |  |
| Previous experience with HRSR assistance (n=154) |  |  |  |  |  |  |  |  | .14 |
| No HRSRs assistance | 131 | 86.8 | 56 | 93.3 | 22 | 84.6 | 53 | 81.5 |  |
| Any HRSRs assistance | 20 | 13.3 | 4 | 6.7 | 4 | 15.4 | 12 | 18.5 |  |
| Discrimination in medical settings (n=150) |  |  |  |  |  |  |  |  | > 0.99 |
| No discrimination | 122 | 83.0 | 47 | 82.5 | 21 | 84.0 | 54 | 83.1 |  |
| Experienced discrimination | 25 | 17.0 | 10 | 17.5 | 4 | 16.0 | 11 | 16.9 |  |
| Trust in healthcare providers (n=150) |  |  |  |  |  |  |  |  | .17 |
| Less than complete trust | 51 | 34.5 | 25 | 42.4 | 9 | 36.0 | 17 | 26.6 |  |
| Complete trust | 97 | 65.5 | 34 | 57.6 | 16 | 64.0 | 47 | 73.4 |  |
| HRSRs status |  |  |  |  |  |  |  |  | .44 |
| No HRSR | 96 | 63.6 | 41 | 68.3 | 14 | 53.9 | 41 | 63.1 |  |
| ≥1 HRSRs | 55 | 36.4 | 19 | 31.7 | 12 | 46.2 | 24 | 36.9 |  |
| Desiring assistance with HRSRs (n=153) |  |  |  |  |  |  |  |  | .01 |
| No | 109 | 72.7 | 51 | 85.0 | 14 | 56.0 | 44 | 67.7 |  |
| Yes | 41 | 27.3 | 9 | 15.0 | 11 | 44.0 | 21 | 32.3 |  |
